# Therapieoptionen beim oligometastasierten Magenkarzinom

**DOI:** 10.1007/s00104-021-01353-5

**Published:** 2021-02-05

**Authors:** Minoa Karin Jung, Katja Ott, Mickael Chevallay, Stefan Paul Mönig

**Affiliations:** 1 Abteilung Viszeral- und Transplantationschirurgie, Chirurgische Klinik, Universitätsklinikum Genf, Rue Gabrielle Perret Gentil 4, 1211 Genf, Schweiz; 2 Chirurgische Klinik, Klinikum Rosenheim, Rosenheim, Deutschland

**Keywords:** S3-Leitlinie, Adenokarzinom des ösophagogastralen Übergangs, Oligometastasierung, Diffuse Metastasierung, Multimodale Therapie, S3 guidelines, Adenocarcinoma of the esophagogastric junction, Oligometastasis, Diffuse metastasis, Multimodal treatment

## Abstract

**Hintergrund:**

Zum Zeitpunkt der Diagnosestellung des Magenkarzinoms haben ca. zwei Drittel der Patienten bereits Metastasen. Wichtig ist es, die Oligometastasierung von der diffus metastasierten Situation abzugrenzen. Die S3-Leitlinie hat die Definition der Oligometastasierung erstmals in die Leitlinie aufgenommen.

**Fragestellung:**

Kann das Überleben von Patienten mit Oligometastasierung mittels Resektion des Tumors und der Metastase kombiniert mit perioperativer Chemotherapie verbessert werden?

**Material und Methoden:**

In dieser Übersichtsarbeit wird die Datenlage der aktuellen Literatur dargestellt.

**Ergebnisse:**

Die holländische Magenkarzinomstudie stellte ein verbessertes medianes Überleben für Patienten mit singulären Metastasen fest, wenn diese reseziert wurden. Aufgrund der Resultate der deutschen AIO-FLOT3-Studie, in der sich das mediane Überleben von Patienten mit Oligometastasen mit multimodaler Therapie verdoppelte, wurde die AIO-FLOT5(RENAISSANCE)-Studie initiiert. Diese randomisiert limitiert metastatische Patienten nach neoadjuvanter Chemotherapie entweder zu Resektion gefolgt von Chemotherapie oder zu definitiver Chemotherapie. Weitere randomisierende Studien untersuchen den Nutzen von Antikörpern und Immun-Checkpoint-Inhibitoren beim lokoregionalen und metastasierten Magenkarzinom mit vielversprechenden Resultaten.

**Diskussion:**

Die Resultate der aktuellen Studien werden zeigen, ob Patienten mit Oligometastasierung von einer multimodalen Therapie mit Resektion profitieren. Die eindeutige Definition der Oligometastasierung, eine Beurteilung des Ansprechens nach neoadjuvanter Chemotherapie und eine realistische Einschätzung der R0-Resektion werden bei der entsprechenden Patientenselektion hilfreich sein.

## Abstract (en)

**Background:**

At the time of diagnosis of gastric cancer approximately one third of patients already have metastases. It is important to differentiate between oligometastasis and the diffuse metastatic situation. For the first time the definition of oligometastasis has been integrated into the German S3 guidelines.

**Objective:**

Can multimodal treatment with tumor resection and metastasectomy combined with perioperative chemotherapy, increase the chances of survival in oligometastatic patients?

**Material and methods:**

In this review article the data situation of the current literature is discussed.

**Results:**

The Dutch D1/D2 trial reported an increased median survival for a subgroup of patients with single metastasis who underwent resection. Multimodal treatment with resection doubled the median survival of oligometastatic patients in the German AIO-FLOT 3 study and as a consequence, the AIO-FLOT 5 (RENAISSANCE) trial was designed. Patients with oligometastatic gastric and esophagogastric junction cancer are randomized after chemotherapy to either undergo resection followed by adjuvant chemotherapy or to undergo definitive chemotherapy. Further randomized trials investigate the benefit of antibodies and immune checkpoint inhibitors in locoregional and advanced metastatic gastric cancer with promising results.

**Conclusion:**

The results of the ongoing randomized trials will show if oligometastatic patients benefit from a multimodal treatment with resection. The clear definition of the oligometastatic state, assessment of the response to neoadjuvant chemotherapy and realistic estimation of the R0 resectability will be useful for patient selection.

## Epidemiologie des oligometastasierten Magenkarzinoms

In Deutschland wird für das Jahr 2020 mit 5400 Neuerkrankungen an Magenkrebs bei Frauen und 8900 bei Männern gerechnet. Ein Drittel der Fälle mit ausreichender Dokumentation ist bereits bei Diagnosestellung metastasiert (Stadium IV; [18]). Wichtig ist, zwischen einer systemisch metastasierten palliativen Erkrankung und einer oligometastasierten, limitiert metastasierten Erkrankung zu unterschieden. Die Therapie der oligometastasierten Erkrankung wurde erstmals in die aktuelle Fassung der deutschen S3-Leitline des Magenkarzinoms aufgenommen [20, 21].

Jedem Chirurgen sind Einzelfälle erinnerlich, wo oligometastasierte Magenkarzinome mit multimodaler Therapie und Resektion kurativ behandelt wurden. Dennoch wird die Chirurgie beim limitiert metastasierten Magenkarzinom aufgrund der vorliegenden Daten sehr kontrovers diskutiert [13, 19, 27]. Bislang fehlt eine exakte Definition für die Möglichkeit kurativer operativer Konzepte, sodass daraus derzeit keine generellen Therapieempfehlungen für das oligometastasierte Magenkarzinom abgeleitet werden können.

## Definition der Oligometastasierung

Basierend auf der AIO-FLOT (Arbeitsgemeinschaft Internistische Onkologie –Fluorouracil, Leucovorin, Oxaliplatin und Docetaxel) 3‑Studie von Al-Batran et al. [6] wurde die Hypothese generiert, dass eine selektierte Subgruppe mit limitierter metastasierter Erkrankung nach Chemotherapie von einer Resektion des Primärtumors und der Metastasen profitieren könnte.

Aus vorliegenden pro- und retrospektiven Studien lassen sich prinzipielle Kriterien definieren [6, 9, 13, 19, 24, 27], bei denen Patienten mit limitiert metastasiertem Magenkarzinom möglicherweise von einer Resektion des Primärtumors und der Metastasen profitieren könnten. Begünstigende Faktoren sind insbesondere die R0-Resektion, einzelne Metastasen, fehlende Peritonealkarzinose und das Ansprechen auf eine systemische Chemotherapie vor der Resektion [8]. Relevante Voraussetzung für die Entscheidung zur Resektion sollte die realistische Möglichkeit der R0-Resektion von Primärtumor und Metastasen in der synchronen Situation bzw. der Metastasen in der metachronen Situation ebenso wie eine vorausgegangene Chemotherapie sein [9, 24, 27]. Der Vorteil einer Kombinationsbehandlung von Chirurgie und Chemotherapie im Vergleich zu einem alleinigen chirurgischen Vorgehen ist durch eine Metaanalyse belegt [27].

Um eine Vergleichbarkeit von Studienergebnissen zu gewährleisten, sollte eine limitierte Metastasierung definiert werden. Für die FLOT5-Studie [3] wurde das limitiert metastasierte Magenkarzinom zukunftsweisend klar definiert (Infobox 1), zusätzlich wird die Einschätzung der Resektabilität zentral überprüft. Diese Definition wurde auch in die aktuelle S3-Leitlinie integriert [20, 21].

### Infobox 1 Definition des limitierten metastatischen Status gemäß der FLOT3-Studie mit Modifikation [3, 20]

- Retroperitoneale Lymphknotenmetastasen (RPLM; z. B. paraaortale, intraaortokavale, parapankreatische oder mesenteriale Lymphknoten)

Hinweis: Ins Duodenum eindringender Magenkrebs und retropankreatische Lymphknoten werden nicht als M1 angesehen.

Als *Oligometastasierung gilt*, wenn nach dem folgenden Schema *maximal ein Organ einbezogen ist, mit oder ohne RPLM*:

- lokalisierte potenziell operable Peritonealkarzinose: Stadium P1 (direkt an das Magenkarzinom angrenzende Peritonealkarzinose oberhalb des Colon transversum) nach der Klassifikation der „Japanischen Magenkrebsforschungsgesellschaft“, d. h. klinisch sichtbare Karzinose des Peritoneums oder Pleura (Cave: >P1-Peritonealkarzinose) sind nicht erlaubt!
- Leber: maximal 5 potenziell resektable metastatische Läsionen
- Lunge: einseitige Beteiligung, potenziell resektabel
- uni- oder bilaterale Krukenberg-Tumoren
- uni- oder bilaterale Nebennierenmetastasen oder
- extraabdominale Lymphknotenmetastasen wie supraklavikuläre oder zervikale Lymphknotenbeteiligung (Virchow-Knoten) oder
- klar lokalisierte Knochenbeteiligung (definiert als innerhalb eines Bestrahlungsfeldes)

## Aktuelle Leitlinien (S3, ESMO, NCCN)

Die aktuellen deutschen S3-Leilinien haben erstmals Empfehlungen zum limitiert metastasierten Magenkarzinom und Adenokarzinom des ösophagogastralen Übergangs aufgenommen. Dort sind die Definition für eine limitierte Metastasierung (Infobox 1) hinterlegt und folgende konsensbasierte Aussagen getroffen worden:

- Eine Resektion von Primärtumor und Metastasen sollte außerhalb von Studien nicht erfolgen.
- Im Einzelfall können erst intraoperativ entdeckte, limitierte Metastasen, wenn R0 resektabel, reseziert werden.
- Patienten mit synchron limitierten Metastasen sollte die Überweisung in eine Klinik mit hoher Fallzahl angeboten werden.

Limitiert metastasierte Patienten sollten an Zentren behandelt werden, wo sie in Studien, wie aktuell die FLOT5-Studie [3], eingeschlossen werden können und Expertise für Magenchirurgie und Metastasenchirurgie besteht. Die zweite Aussagen wurde analog der Leitlinien des Ösophaguskarzinoms [23] formuliert und erleichtert die intraoperative Entscheidungsfindung bei im Staging okkult gebliebener Metastasierung.

Die Resektion von Primärtumor und Metastasen außerhalb von Studien wird nicht empfohlen

In den aktuellen ESMO(European Society for Medical Oncology)-Guidelines [26] ist eine Resektion des Primärtumors in der metastasierten Situation nicht vorgesehen. Allerdings wird angemerkt, dass eine Subgruppe nach gutem Ansprechen auf die systemische Therapie möglicherweise für eine Resektion infrage kommen könnte. Bis weitere belastbare Daten vorliegen, wird in den ESMO-Guidelines sowohl die Gastrektomie als auch die Metastasenresektion in der metastasierten Situation als „experimentell“ eingeschätzt. Getrennt wird dezidiert die Peritonealkarzinose angesprochen. Zytoreduktive Chirurgie und hypertherme intraperitoneale Chemotherapie wird an nichtasiatischen Patienten ebenfalls außerhalb von Studien nicht empfohlen, parallel zur deutschen S3-Leitlinie.

Die NCCN(National Comprehensive Cancer Network)-Guidelines [1] schlagen eine palliative Therapie („best supportive care“, systemische Chemotherapie, Studienteilnahme) für lokal nichtresektable oder systemisch metastasierte Karzinome oder Rezidive vor. Chirurgie wird als eine Option bei lokalisierten Rezidiven bei fitten Patienten genannt.

Prinzipiell ist die Resektion von Primärtumor und Metastasen außerhalb von Studien aktuell in keiner Leitlinie als Behandlungsoption empfohlen.

## Chirurgische Therapieoptionen

Mehrere retrospektive Studien zum Magenkarzinom mit Patienten mit Stadium IV konnten ein verbessertes Überleben nach Chemotherapie und Gastrektomie im Vergleich zur alleinigen Chemotherapie aufzeigen [9, 10, 19, 24]. Bei diesen retrospektiven Studien muss immer ein Selektionsbias in der Gruppe mit Resektion berücksichtigt werden.

Bereits in der holländische Magenkarzinomstudie zur Wertigkeit der D2-Lymphadenektomie konnte bei Patienten unter 70 Jahren mit singulärer Metastasenlokalisation ein Überlebensvorteil bei Resektion aufgezeigt werden [14, 15].

### REGATTA-Studie

Die erste prospektiv randomisierte Studie, welche den Fokus auf den möglichen Vorteil der primären Chirurgie bei Patienten mit fortgeschrittenem Magenkarzinomen mit Oligometastasierung legte, war die 2016 publizierte REGATTA-Studie [13]. Diese international angelegte asiatische Studie schloss 175 Patienten mit fortgeschrittenem Magenkarzinom und Oligometastasierung entweder der Leber, des Peritoneums oder paraaortaler Lymphknoten außerhalb der D3-Dissektion ein. Die Patienten wurden randomisiert zu entweder definitiver Chemotherapie mit S‑1 und Cisplatin ohne Resektion oder Gastrektomie mit D1-Lymphknotendissektion gefolgt von adjuvanter Chemotherapie als Erstlinientherapie. Das Gesamtüberleben nach 2 Jahren war 31,7 % für Patienten mit alleiniger Chemotherapie und 25,1 % für Patienten mit Gastrektomie gefolgt von adjuvanter Chemotherapie (*p* = 0,66).

Aufgrund einer durch eine Zwischenanalyse als gering eingeschätzten Wahrscheinlichkeit eines verbesserten Überlebens in der Gastrektomiegruppe, wurde die Patientenrekrutierung vorzeitig abgebrochen. Die Gastrektomie in der oligometastasierten Situation wurde aufgrund dieser Ergebnisse nicht empfohlen, sodass in Asien die alleinige Chemotherapie weiterhin den Goldstandard auch beim limitiert metastasierten Magenkarzinom darstellt.

REGATTA klärt nicht die Verbesserung des Überlebens bei R0-Resektion der Metastasen

Aus Sicht der Autoren ist jedoch von entscheidender Bedeutung, dass in der REGATTA-Studie die Metastasen in situ belassen wurden und lediglich eine D1-Lymphknotendissektion der perigastrischen Lymphknoten durchgeführt wurde. Durch dieses Studiendesign konnte somit die aktuell relevante Fragestellung der Verbesserung des Überlebens bei R0-Resektion der Metastasen und des Primärtumors nicht geklärt werden. Zudem wurden im chirurgischen Arm weniger Patienten chemotherapiert als im konservativen Arm, da die adjuvante Chemotherapie aufgrund verminderter Compliance und Gewichtsverlust insbesondere nach totaler Gastrektomie nur in geringerer Zykluszahl verabreicht werden konnte. Die Subgruppe der Patienten mit distalem Magenkarzinom und zumeist subtotaler Gastrektomie konnte signifikant häufiger adjuvant chemotherapiert werden und zeigte ein besseres Überleben als die Gruppe mit alleiniger Chemotherapie.

### FLOT3-Studie

Die deutsche FLOT3-Studie untersuchte das Überleben von 252 Patienten mit Magen- und Übergangskarzinomen nach Verabreichung einer neoadjuvanten Chemotherapie mit mindestens 4 Zyklen FLOT gefolgt von Resektion und schloss auch Patienten mit Metastasierung ein [6]. Sechzig Patienten wurden als limitiert metastasiert beurteilt und 36 dieser Patienten erhielten nicht nur eine Resektion des Primärtumors, sondern auch eine Entfernung der Metastase. Es handelte sich bei den Metastasenresektionen bei 18 Patienten um retroperitoneale Lymphknoten, bei 6 Patienten um Lebermetastasen, bei weiteren 6 Patienten um Lungenmetastasen, bei 2 Patienten um lokale Peritonealkarzinomatose und bei 4 Patienten um andere Metastasen.

Das mediane Gesamtüberleben der oligometastasierten Patienten, welche eine neoadjuvante Chemotherapie und eine Resektion erhielten war 31,1 Monate, während sich das Überleben der oligometastasierten Patienten, welche eine Chemotherapie ohne Chirurgie erhielten, auf 15,9 Monate belief. Zu erwähnen bleibt, dass die Patienten deren Oligometastasierung die retroperitonealen Lymphknoten betraf, die beste Prognose innerhalb der metastasierten Patienten hatten. Verantwortlich für die gute Prognose der operierten oligometastasierten Patienten war eine R0-Resektionsrate von 80,6 %. Auch bei diesen Ergebnissen muss ein Selektionsbias beachtet werden.

### FLOT5-Studie

**Figure 1:**
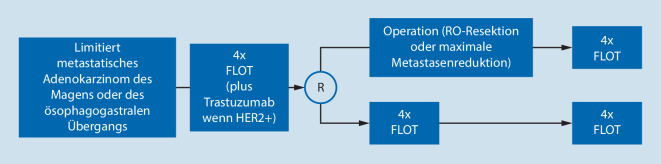

In Anbetracht dieser Resultate wurde die multizentrische, prospektive RENAISSANCE-FLOT5(AIO-CAOGI[Chirurgischen Arbeitsgemeinschaft für den Oberen Gastrointestinaltrakt])-Studie initiiert [3]. In dieser Studie werden Patienten mit limitiert metastatischen Magen- und Adenokarzinomen des ösophagogastralen Übergangs (AEG) eingeschlossen. Die Patienten erhalten initial 4 Zyklen FLOT, dieses Regime wird im Falle von HER2-Protein-exprimierenden Karzinomen mit Trastuzumab kombiniert. Patienten ohne Progress während der Chemotherapie werden randomisiert entweder zur Resektion des Primärtumors und der Metastase gefolgt von weiteren 4 Zyklen Chemotherapie oder zur definitiver Chemotherapie mit 8 bis 12 Zyklen (Abb. 1). Aktuell wurden 143 Patienten rekrutiert und 108 (von 176) randomisiert.

Eine Auswahl randomisierter Studien mit Patienten mit metastatischem Magen- oder gastroösophagealen Übergangskarzinom findet sich in Tab. 1.

**Table 1:** 

Studie	Behandlung	Gesamtzahl Patienten mit Metastasen und/oder irresektablem Tumor	Überleben Kontrollgruppe ohne Resektion	Überleben Interventionsgruppe mit Resektion
DGCT [14]	Keine Chemotherapie ±Tumorresektion	*n* = 285	*n* = 129 Medianes Gesamtüberleben 5,4 Monate	*n* = 156 Medianes Gesamtüberleben 8,1 Monate
REGATTA [13]	S‑1 + Cisplatin adjuvant ±Tumorresektion	*n* = 175	*n* = 86 Medianes Gesamtüberleben 16,6 Monate	*n* = 89 Medianes Gesamtüberleben 14,3 Monate
FLOT3-AIO [6]	FLOT Perioperativ ±Tumorresektion plus Metastasektomie	*n* = 187	*n* = 24 (oligometastatisch) Medianes Gesamtüberleben 15,9 Monate	*n* = 36 (oligometastatisch) Medianes Gesamtüberleben 31,1 Monate
FLOT5-AIO [3]	FLOT Perioperativ ±Tumorresektion plus Metastasektomie	*n* = 176	Rekrutierend	Rekrutierend
**Studie**	**Behandlung**	**Gesamtzahl Patienten mit Metastasen und/oder irresektablem Tumor**	**Überleben Kontrollgruppe Chemotherapie ohne Antikörpertherapie**	**Überleben Interventionsgruppe Chemotherapie mit Antikörpertherapie**
ToGA [7]	5‑FU (oder Capecitabin) +Cisplatin ±Trastuzumab	*n* = 594 (HER2-positiv)	*n* = 296 Medianes Gesamtüberleben 11,1 Monate	*n* = 298 Medianes Gesamtüberleben 13,8 Monate

*FLOT* Fluorouracil, Leucovorin, Oxaliplatin und Docetaxel; *5‑FU* 5‑Fluorouracil

## Chemotherapie

Es besteht ein weitreichender Konsens, dass eine Resektion des oligometastasierten Magenkarzinoms nicht ohne Chemotherapie stattfinden sollte. In Europa hat sich analog zum lokal fortgeschrittenen Karzinom ein perioperatives Konzept wegen der schlechterer Compliance und Toleranz der adjuvanten Chemotherapie herauskristallisiert [11]. Auch bezüglich des optimalen Chemotherapieregimes gibt es weltweit unterschiedliche Konzepte.

FLOT ist beim lokal fortgeschrittenen Magen- und Übergangskarzinom Behandlungsstandard

In Europa wird aktuell das FLOT-Regime als Standard für die perioperative/neoadjuvante Therapie eingesetzt. Die randomisierte FLOT4-Studie mit 716 Patienten konnte für FLOT eine signifikant höhere komplette histopathologische Regression und höhere R0-Resektionsrate im Vergleich zu ECF (Epirubicin, Cisplatin, 5‑Fluorouracil) und ECX (Epirubicin, Cisplatin, Capecitabin) aufzeigen [5]. FLOT führte im Vergleich zu ECF/ECX bei Patienten mit Magen- und Übergangskarzinomen ab dem klinischen Stadiums cT2 und/oder klinischem Lymphknotenbefall cN^+^ bei resezierbaren Tumoren zu einem verbesserten medianem Gesamtüberleben von 50 Monaten vs. 35 Monaten (*p* = 0,012). FLOT ist damit zum neuen empfohlenen Behandlungsstandard beim lokal fortgeschrittenen Magen- und Übergangskarzinom geworden.

## Metastasiertes Magenkarzinom

### Erstlinienchemotherapie bei fortgeschrittenem/metastasiertem Magenkarzinom

Beim fortgeschrittenen, nichtresezierbaren oder metastasierten Magenkarzinom erhöht die Chemotherapie das Überleben und wird bei erhaltenem Allgemeinzustand empfohlen [20]. Erhöhtes Alter stellt dabei keine Kontraindikation dar. Als Erstlinienchemotherapie wird in der Regel ein Duo mit einem Platinum und Fluoropyrimidin empfohlen [1, 26], wobei bei älteren Patienten Oxaliplatin statt Cisplatin in Kombination mit 5‑Fluoruracil zu weniger Nebenwirkungen zu führen scheint [4].

Taxanbasierte Dreierkombinationen mit Cisplatin/Oxaliplatin, 5‑Fluorouracil und Docetaxel sind ebenfalls möglich, führen aber häufiger zu unerwünschten Nebenwirkungen (Neutropenie) und sind daher eher jüngeren Patienten in gutem Allgemeinzustand mit hoher Tumorlast vorbehalten [28].

### Erstlinienchemotherapie kombiniert mit Antikörpertherapie bei fortgeschrittenem Magenkarzinom

Etwa 17–20 % der Magenkarzinompatienten zeigen eine Überexpression des HER2-Proteins [22]. In der palliativen Therapie nichtoperabler Patienten hat sich in der ToGA-Studie (Trastuzumab in combination with chemotherapy versus chemotherapy alone for treatment of HER2-positive advanced gastric or gastro-oesophageal junction cancer) ein Überlebensvorteil gezeigt, wenn Patienten mit HER2-positiven Magenkarzinomen zusätzlich zu Fluoropyrimidin und Cisplatin den Antikörper Trastuzumab erhielten [7]. Das mediane Gesamtüberleben war 13,8 Monate für Patienten mit Chemotherapie plus Trastuzumab vs. 11,1 Monate für Patienten mit alleiniger Chemotherapie (*p* = 0,0046; [7]).

Vor dem Einsatz einer palliativen Chemotherapie bei metastasierten Karzinomen soll der HER2-Status bestimmt werden und bei HER2-Überexpression zusätzlich zur platin-/fluoropyrimidinbasierten Chemotherapie Trastuzumab als Erstlinientherapie verabreicht werden [20]. Aktuell wird ein neoadjuvantes Konzept für HER-2-positive Tumoren in der AIO PETRARCA-FLOT6-Studie [16] überprüft. Die auf dem ESMO-Kongress 2020 vorgestellten Ergebnisse sind vielversprechend mit signifikant erhöhten kompletten pCR(pathologische Komplettremission)-Raten nach Trastuzumab/Pertuzumab-Therapie in Kombination mit FLOT (Tab. 2).

**Table 2:** 

	Behandlung	Gesamtzahl Patienten mit resektablem Tumor (≥cT2 oder cN+)	Überleben Kontrollgruppe Chemotherapie ohne Zusatztherapie	Überleben Interventionsgruppe Chemotherapie mit Zusatztherapie
PETRARCA [16]	FLOT perioperativ ± Trastuzumab/Pertuzumab plus Resektion	*n* = 81 (HER2-positiv)	*n* = 41 Gesamtüberleben nach 2 Jahren 77 % pCR-Rate 12 %	*n* = 40 Gesamtüberleben nach 2 Jahren 84 % pCR-Rate 35 %
DANTE [2]	FLOT perioperativ ± Atezolizumab plus Resektion	*n* = 295	Rekrutierend	Rekrutierend

*FLOT* Fluorouracil, Leucovorin, Oxaliplatin und Docetaxel; *pCR* pathologische Komplettremission

### Immunotherapie bei fortgeschrittenem Magenkarzinom

Die Immunotherapie hat Vorteile gezeigt bei Tumorprogression nach abgeschlossener Zweit- oder Drittlinienchemotherapie.

Der monoklonale PD-L1-Inhibitor Nivolumab führte in einer randomisierten Studie im Vergleich zu Placebo zu einem signifikant verbesserten Gesamtüberleben bei mehrfach vorbehandelten asiatischen Patienten [17]. Ein weiterer PD-L1-Inhibitor, Pembrolizumab, zeigte ebenfalls ein verbessertes Gesamtüberleben bei Patienten, die mindestens schon eine Zweitlinienchemotherapie erhalten hatten [12].

Der Stellenwert einer neoadjuvanten Immunotherapie (Atezolizumab) in Kombination mit FLOT wird aktuell in der AIO-DANTE-FLOT8-Studie evaluiert [2].

## Zukunftsaussichten

Die vorgestellten Studienergebnisse veranlassen verschiedene Gruppen, die Gabe der Antikörpertherapie und Immunotherapie in Kombination mit Chemotherapie auch auf die Erstlinientherapie des resezierbaren, des lokoregional fortgeschrittenen und des oligometastatischen Magenkarzinoms auszuweiten.

Der Stellenwert einer neaodjuvanten Immunotherapie (Atezolizumab) in Kombination mit FLOT wird aktuell in der AIO-DANTE-FLOT8-Studie evaluiert.

Die Gabe von Trastuzumab und Pertuzumab in Kombination mit Chemotherapie beim resezierbaren Magenkarzinom wird zurzeit in der EORTC-1203-INNOVATION-Studie untersucht [29]. Die EORTC-1707-VESTIGE-Studie untersucht die adjuvante Gabe von Immunotherapie mit Nivolumab und Ipilimumab bei nach neoadjuvanter Therapie nodal positiven Karzinomen und bei positiven Sicherheitsabständen vergleichend zu der adjuvanten Gabe des gleichen Chemotherapieregimes, welches bereits neoadjuvant gegeben wurde [25]. Die laufende RENAISSANCE-Studie kombiniert aktuell bereits die adjuvante FLOT-Chemotherapie mit Trastuzumab bei HER2-exprimierenden oligometastatischen Karzinomen.

Das Ergebnis der RENAISSANCE-Studie wird hoffentlich zeigen, ob die Resektion integraler Bestandteil der multimodalen Therapie des Magen sein könnte. Ziel der Zukunft wird bleiben, Subgruppen (durch prätherapeutische molekulare Stratifizierung, Responseevaluation nach Therapie und realistische Einschätzung der R0-Resektablilität) zu definieren, die von einer Resektion von Primärtumor und Metastasen in der oligometastasierten Situation profitieren.

Hierzu hat sich auf Initiative unserer holländischen Kollegen eine europäische Gruppe zusammengefunden (OMEC, OligoMetastasis in Esophago-gastric Cancer).

## Fazit für die Praxis

- Die Resultate der aktuell rekrutierenden randomisierten Studien mit oligometastasierten Patienten werden zeigen, ob Patienten von einer Resektion kombiniert mit perioperativer Chemotherapie profitieren.
- Die klare Definition der Oligometastasierung, ein Restaging nach präoperativer Chemotherapie mit Beurteilung des Ansprechens und eine realistische Einschätzung der R0-Resektion werden in die Entscheidung dieses „kurativen“ Therapieansatzes bei ausgewählten oligometastatischen Patienten im interdisziplinären Tumorboard einfließen.
- Oligometastasierte Patienten sollten diagnostisch für ein optimales Staging und zur Klassifikation der Peritonealkarzinose laparoskopiert und HER2-getestet werden.
- Eine histologische Sicherung der Metastasen ist wegen relevanter therapeutischer Konsequenzen und Vergleichbarkeit erforderlich.
- Die Kriterien für ein oligometastasiertes Magenkarzinom sind anzuwenden (Infobox 1).
- Standard für das oligometastasierte Magenkarzinom ist derzeit die palliative Chemotherapie.
- Der Studieneinschluss oligometastasierter Patienten in Zentren (aktuell FLOT 5 rekrutierend) wird empfohlen.
